# Glutamate and GABA in Vestibulo-Sympathetic Pathway Neurons

**DOI:** 10.3389/fnana.2016.00007

**Published:** 2016-02-08

**Authors:** Gay R. Holstein, Victor L. Jr. Friedrich, Giorgio P. Martinelli

**Affiliations:** ^1^Department of Neurology, Icahn School of Medicine at Mount SinaiNew York, NY, USA; ^2^Department of Neuroscience, Icahn School of Medicine at Mount SinaiNew York, NY, USA; ^3^Department of Anatomy/Functional Morphology, Icahn School of Medicine at Mount SinaiNew York, NY, USA

**Keywords:** vestibular, blood pressure, otolith, sympathetic nerve activity, galvanic vestibular stimulation

## Abstract

The vestibulo-sympathetic reflex (VSR) actively modulates blood pressure during changes in posture. This reflex allows humans to stand up and quadrupeds to rear or climb without a precipitous decline in cerebral perfusion. The VSR pathway conveys signals from the vestibular end organs to the caudal vestibular nuclei. These cells, in turn, project to pre-sympathetic neurons in the rostral and caudal ventrolateral medulla (RVLM and CVLM, respectively). The present study assessed glutamate- and GABA-related immunofluorescence associated with central vestibular neurons of the VSR pathway in rats. Retrograde FluoroGold tract tracing was used to label vestibular neurons with projections to RVLM or CVLM, and sinusoidal galvanic vestibular stimulation (GVS) was employed to activate these pathways. Central vestibular neurons of the VSR were identified by co-localization of FluoroGold and cFos protein, which accumulates in some vestibular neurons following galvanic stimulation. Triple-label immunofluorescence was used to co-localize glutamate- or GABA- labeling in the identified VSR pathway neurons. Most activated projection neurons displayed intense glutamate immunofluorescence, suggestive of glutamatergic neurotransmission. To support this, anterograde tracer was injected into the caudal vestibular nuclei. Vestibular axons and terminals in RVLM and CVLM co-localized the anterograde tracer and vesicular glutamate transporter-2 signals. Other retrogradely-labeled cFos-positive neurons displayed intense GABA immunofluorescence. VSR pathway neurons of both phenotypes were present in the caudal medial and spinal vestibular nuclei, and projected to both RVLM and CVLM. As a group, however, triple-labeled vestibular cells with intense glutamate immunofluorescence were located more rostrally in the vestibular nuclei than the GABAergic neurons. Only the GABAergic VSR pathway neurons showed a target preference, projecting predominantly to CVLM. These data provide the first demonstration of two disparate chemoanatomic VSR pathways.

## Introduction

Homeostatic control of blood pressure is mediated by the baroreflex, a highly effective closed-loop negative feedback pathway responsible for maintaining stable blood pressure (Hall, 2016). The baroreflex utilizes inputs from carotid and aortic baroreceptors that are conveyed to the solitary nucleus (Spyer, 1981) and from there to the caudal ventrolateral medulla (CVLM). The CVLM sends both excitatory and inhibitory projections to the rostral ventrolateral medulla (RVLM), which in turn provides critical descending input to the preganglionic neurons in the spinal cord that control sympathetic nerve activity including the innervation of vascular smooth muscle (Blessing, 1988; Holstege, 1989; Guyenet, 1990; Stocker et al., 1997; Chan and Sawchenko, 1998; Pilowsky and Goodchild, 2002; Dampney et al., 2003; Schreihofer et al., 2005; Heesch et al., 2006). The baroreflex pathway is polysynaptic, poorly myelinated, has a relatively long latency and is solely reactive, modulating vasoconstriction and heart rate in response to a prior cardiovascular vicissitude (Yates et al., 2014a) such as a drop in blood pressure. More direct and rapid modulation of blood pressure in response to head movements and other postural adjustments is achieved through interactions between vestibular end organ-activated neurons in the vestibular nuclei and presympathetic brainstem cell groups, including RVLM and CVLM (Yates and Bronstein, 2005). These vestibulo-sympathetic reflex (VSR) pathways provide a proactive mechanism for initiating blood redistribution in the body during movement or changes in posture in order to assure consistent cerebral perfusion (Yates, 1996; Kerman et al., 2000b) regardless of head position (Yates and Miller, 1994; Woodring et al., 1997; Balaban and Yates, 2004; Abe et al., 2009; Nakamura et al., 2009).

The first experimental demonstration of the VSR was published in 1974 (Doba and Reis, 1974). That study reported blood pressure responses to whole body nose-up tilt before and after bilateral vestibular nerve section in cats. Since blood pressure dropped more precipitously after the deafferentation, the investigators concluded that the lesion caused orthostatic intolerance, suggesting a role for the vestibular system in blood pressure modulation. Since then, studies in several experimental models, including cats, rabbits and rats, utilizing a variety of physiological vestibular stimuli such as caloric stimulation, head rotation, whole body tilt, horizontal and vertical linear acceleration, centrifugation, off-vertical-axis rotation, Ferris Wheel rotation and spaceflight have contributed to our understanding of the VSR (Kaufman et al., 1992; Marshburn et al., 1997; Gustave Dit Duflo et al., 2000; Saxon et al., 2001; Chen et al., 2003; Fuller et al., 2004; Kaufman, 2005; Zhang et al., 2005; Lai et al., 2006, 2008; Tse et al., 2008; Abe et al., 2009; Baizer et al., 2010; Cai et al., 2010). Additional information has been gleaned from studies utilizing electrical stimulation of the vestibular end organs and/or nerve, including galvanic vestibular stimulation (GVS).

Sinusoidally modulated GVS (sGVS) involves the passage of low frequency direct current (1–4 mA) across the labyrinths delivered through electrodes inserted under the skin over the mastoid processes (Bent et al., 2006; Curthoys, 2010; Hammam et al., 2011). The stimulus is experimentally advantageous for studies of the VSR because it is minimally invasive, does not cause shifts in body fluids, and is unlikely to activate neck proprioceptors or non-vestibular abdominal graviceptors (Yates et al., 2014a). Stimulation at the cathode specifically activates the vestibular nerve fibers, particularly those with irregular spontaneous discharge rates (Goldberg et al., 1984; Minor and Goldberg, 1991), while afferents at the anode are inhibited (Fitzpatrick and Day, 2004). S-GVS appears to exert a more robust and prolonged effect on the otolith system than on the semicircular canal pathways, and is a highly effective stimulus for modulating sympathetic nerve activity and blood pressure in humans and experimental animals (Courjon et al., 1987; Ray et al., 1998; Abe et al., 2008b, 2009; Cohen et al., 2011b; Yates et al., 2014a).

Despite a large body of research demonstrating the existence and functionality of the VSR, relatively little is known about the specific connectivity and chemical anatomy of neurons involved in the vestibular modulation of blood pressure. Our laboratory has previously demonstrated that there is a direct projection from the caudal vestibular nuclei to RVLM and CVLM (Holstein et al., 2011). This work also documented the cytology of vestibular neurons participating in these pathways, and the morphology of their axons and terminals. Subsequently, we utilized sGVS to modulate blood pressure, and then visualized cFos protein in vestibular neurons activated by the stimulation (Holstein et al., 2012). We observed that sGVS-activated neurons were present throughout the rostro-caudal extent of the vestibular nuclear complex, and were particularly concentrated in parts of the spinal (SpVN), medial (MVN) and superior vestibular nuclei. Based on a qualitative assessment, it was estimated that approximately one third of the cFos-positive vestibular neurons showed intense glutamate immunofluorescence. However, vestibular neurons activated by GVS and containing the immediate early gene *cfos* could participate in one or more of the vestibular effector pathways that are particularly sensitive to otolith stimulation, including otolith-ocular reflexes, ascending vestibulo-autonomic pathways to limbic-related regions, as well as the descending projections to RVLM and CVLM involved in homeostatic functions. In order to identify the vestibular neurons specifically involved in this latter pathway, we combined cFos detection of sGVS-activated neurons with retrograde tract tracing from RVLM or CVLM, and mapped the locations of the double-labeled activated projection neurons of the VSR (Holstein et al., 2014). These maps indicated that VSR neurons projecting to RVLM and CVLM are only present in the caudal half of MVN and SpVN, a region that is significantly more discrete than the vestibular areas identified by cFos localization alone. The present study was conducted to identify glutamate and GABA immunofluorescence associated with the activated projection neurons contributing to the descending vestibulo-autonomic projection, as a first step toward specifying the chemoanatomic phenotype of VSR pathway neurons. These two amino acids were selected for this study because of their well-documented roles in other vestibular efferent pathways.

## Materials and Methods

All experiments were conducted in accordance with the NRC Guide for the Care and Use of Laboratory Animals (8th Edition, 2011), and were approved by the Institutional Care and Use Committee of the Icahn School of Medicine at Mount Sinai, NY, USA. Data were obtained from 23 adult male Long-Evans rats (Harlan Laboratories, MA, USA) weighing 350–450 g. Ten of these animals received injections of the retrograde tracer Fluorogold (Fluorochrome, LLC, Denver, CO, USA) in RVLM, seven had FluoroGold injections in CVLM, and three received injections of the anterograde tracer *Phaseolus vulgaris* leucoagglutinin (Pha-L; Vector Labs; Cat. #L-1110; Burlingame, CA, USA) in the caudal vestibular nuclear complex. Sections from three additional rats in which the retrograde tracer injections spanned parts of both RVLM and CVLM were used exclusively for corroborative qualitative observations.

### Retrograde Tracer Injections

FluoroGold was utilized because of its high sensitivity, low probability of uptake by fibers of passage and lack of concomitant anterograde transport (Raju and Smith, 2006; Schofield, 2008). Rats were anesthetized with isoflurane (4% induction, 2% maintenance), shaved and placed in a computer-assisted stereotaxic frame (Leica *Angle Two*, Leica Microsystems, St. Louis, MO, USA). Homeothermy was maintained by resting the animals on a homeothermic pad regulated by the feedback from a rectal thermometer. Ophthalmic ointment was used to keep the eyes moist, and one dose of the analgesic Buprenex (Reckitt Benckiser Pharmaceuticals; Richmond, VA, USA) was administered preemptively (0.05 mg/kg, SQ). After draping the animal for aseptic surgery, the head and neck were disinfected with Povidone and a midline incision was made from the top of the skull to the C1 vertebra. The atlanto-occipital membrane was exposed by blunt dissection and retraction of the neck muscles. A glass pipette (tip OD ~20–25 μm) filled with 2% FluoroGold dissolved in saline was mounted on the *Angle Two* dorsoventral drive (DV) tilted 45° over the horizontal plane. The target coordinates for CVLM (ML: ±2.2; AP: −12.80; DV: −10.0) or RVLM (ML: ±2.34; AP: −12.24; DV: −10.21; Paxinos and Watson, 2009) were entered in the computer, and the pipette tip was positioned at Bregma. The *Angle Two* AP and ML drives were then adjusted, and the pipette was advanced under computer guidance toward the brainstem target via a small burr hole (~2 mm dia.) drilled slightly above the atlanto-occipital membrane at ML ± 2.3 mm. The tracer was iontophoresed at +5 μA for 10 min (7 s on, 7 s off). The pipette was left in place for 2–3 min after the conclusion of the iontophoresis, and then slowly withdrawn. The neck muscles were sutured with interrupted stitches and the skin was closed with surgical clips. Rats received 3–4 ml of sterile saline SQ at the end of the procedure. Analgesics were administered twice daily for 3 days after surgery (Buprenex; 0.05 mg/kg; SQ). The animals were allowed to recover for 10–14 days before the terminal experiment was performed.

### Anterograde Tracer Injections

The anesthesia and surgical preparation for these injections were the same as those described above. A small amount of occipital bone was removed with fine rongeurs to allow the glass pipette to reach the vestibular nuclei from a dorsal approach at an angle of 22° from the horizontal plane. The glass pipette (tip OD ~10–15 μm) was initially zeroed on the obex (*calamus scriptorius*) and then moved 0.45 mm caudally and 0.8–1.0 mm laterally. After raising 1.4–1.5 mm (off-vertical movement), the pipette was advanced 2.5–2.8 mm into the caudal vestibular nuclei. Rats received a 15 min iontophoretic injection (7 s on/7 s off; 5 μA) of 2% Pha-L dissolved in 0.1 M phosphate buffer (PB; pH 8.0). The pipette was withdrawn 2–5 min after the injection. The incision closure and postoperative treatment were identical to that described above. The animals were allowed to recover for 10–14 days before the terminal experiment was performed.

### sGVS

Animals anesthetized with isoflurane (4% induction; 2% maintenance) were kept on a homeothermic pad. Ag/AgCl needle electrodes (BAK) connected to a computer-controlled current stimulator (Cohen et al., 2011a) were inserted bilaterally under the skin over the mastoid processes. An individual sGVS stimulus comprised five cycles of binaural current (2 mA, 0.025 Hz). This stimulus was repeated five times with 3 min between repetitions. Rats were then allowed to recover from the anesthesia, and were euthanized 90 min after the cessation of the last sGVS stimulus.

### Tissue Harvesting and Processing

#### Perfusion, Fixation and Tissue Sectioning

Rats anesthetized with isoflurane (as above) were perfused transcardially 90 min after the sGVS stimulation, a time point of peak c-Fos protein accumulation in most experimental paradigms (Kovács, 2008; Durchdewald et al., 2009). In addition, our previous studies demonstrated that a time interval of 90 min after sGVS or tilt stimulation and euthanasia is sufficient for robust cFos protein accumulation in vestibular neurons (Holstein et al., 2012, 2014). The initial perfusion with 100 ml of 37°C 10 mM phosphate buffered saline (PBS) was followed by 500 ml of 4% paraformaldehyde/0.2% glutaraldehyde fixative in 0.1M PB (pH 7.4) at room temperature. Brains were harvested immediately after perfusion, cut into blocks using an adult rat brain coronal matrix (Ted Pella, Inc.; Redding, CA, USA), and stored at 4°C in PBS with 0.02% NaN_3_. A vibrating microtome was used to cut the block containing the vestibular nuclear complex and ventrolateral medullary region into 50 μm serial sections. These sections (~120 per animal) were stored in PBS containing 0.02% NaN_3_ at 4°C.

#### Anatomical Localization

The locations of the four principal vestibular nuclei were determined in each tissue section by comparing the anatomical landmarks on the dorsal aspect of the tissue with a standard stereotaxic atlas (Paxinos and Watson, 2005). Although this atlas was made using sections from Wistar rats, and the present study utilized the Long-Evans strain, we did not observe any significant neuroanatomical differences in the caudal vestibular nuclei, RVLM or CVLM of the two strains. It is worth noting that the *Angle Two* injection guidance system also utilizes Paxinos and Watson coordinate space, and our success rate in targeting the structures of interest is ~80%. This suggests that the Paxinos and Watson stereotaxic atlas coordinates are sufficient for the level of resolution relevant to this study.

Similarly, the boundaries of RVLM and CVLM were determined by comparing the structures on the ventral aspect of the tissue sections with published maps and atlases of those regions (Paxinos and Watson, 2005, 2009; Card et al., 2006; Bourassa et al., 2009; Goodchild and Moon, 2009). Based on the most conservative estimates from these maps, RVLM was localized to a 1 mm rostrocaudal region extending from approximately 11.8–12.8 mm caudal to Bregma. The other dimensions of RVLM were determined by a triangle with the ventral aspect of the medulla 1.4 and 2.2 mm lateral to the midline as two of the points and nucleus ambiguus (pars compacta) at the apex. The CVLM region, located 12.8–13.6 mm caudal to Bregma, was similarly defined according to anatomical coordinates. Both regions correspond well with functional and physiological maps of RVLM and CVLM (Goodchild and Moon, 2009). The locations of RVLM and CVLM were further verified in our experimental animals using tyrosine hydroxylase and GABA immunostaining of representative sections through these regions from the three Long-Evans rats with Pha-L injections in the vestibular nuclei, as previous demonstrated (Holstein et al., 2011).

#### Primary Antibodies

Individual tissue sections were immunostained using one of the following primary antibody combinations: (1) anti-cFos, -FluoroGold and -glutamate; (2) anti-cFos, -FluoroGold and -GABA; (3) anti-Pha-L, -glutamate and -vesicular glutamate transporter (VGluT)-1; (4) anti-Pha-L, -glutamate and -VGluT-2; and (5) anti-Pha-L, -glutamate and -VGluT-3. Antibody sources and descriptions are shown in Table 1. The rationale for their use, staining protocols, and controls are described below.

**Table 1:** **Primary antibodies used in this study**.

Antigen	Host and Type	Immunogen	Source	Working dilutions
FluoroGold	Rabbit polyclonal	Fluorogold	Millipore; Cat. # AB153	1:400 (IMF); 1:5000 (DAB)
c-Fos	Rabbit polyclonal	A peptide mapping within an internal region of human c-Fos	Santa Cruz Biotech; Cat. # Sc-253	1:500
Pha-L	Rabbit polyclonal	Pha-L (E + L), unconjugated	Vector Labs; Cat#AS-2300	1:1000 (DAB); 1:100 (IMF)
Glutamate	Mouse monoclonal IgG1	Glutamate-BSA	Our laboratory; Clone 215B2 Hybridoma supernatant	1:5
GABA	Mouse monoclonal IgG1	GABA-BSA	Our laboratory; Clone C6B6 Hybridoma supernatant	1:20
VGluT-1	Mouse monoclonal IgG1	Fusion protein amino acids 493-560 (cytoplasmic C-terminus) of rat VGlut1	NeuroMab Antibodies; Cat # 75-066	1:10
VGluT-1	Rabbit polyclonal	Fusion protein amino acids 493-560	Synaptic Systems; Cat #135-302	1:500
VGluT-2	Mouse monoclonal IgG1	Fusion protein amino acids 501-582	NeuroMab Antibodies; Cat # 75-067	1:10
VGluT-2	Rabbit polyclonal	Strep-Tag^®^ fusion protein of rat VGLUT 2 (amino acids 510-582)	Synaptic Systems; Cat #135-402	1:800
VGluT-3	Rat monoclonal; IgG1	Fusion protein amino acids 546-588	NeuroMab Antibodies; Cat # 75-073	1:10

We previously evaluated several commercial antibodies against c-Fos including a rabbit polyclonal antiserum directly conjugated with AlexaFluor 488 (Santa Cruz Biotechnology, Cat. # sc-253 AF488) and two unconjugated rabbit polyclonal antisera (Santa Cruz Biotechnology, Cat.# sc-253; Calbiochem, Cat.# PC38; Holstein et al., 2014). Although the three reagents stained the same vestibular regions and cell types, the unlabeled polyclonal sera provided more robust labeling in multiple label immunofluorescence staining experiments. To assess non-specific staining, control tissue sections were exposed to a mixture of the rabbit polyclonal antibody preabsorbed with blocking peptide (Santa Cruz Biotechnology; Cat.# sc-253P); no immunolabeling was apparent in these sections. Previous studies in our laboratory have demonstrated that cFos-peroxidase immunolabeling in mock (non)-sGVS-stimulated rats comprises less than three neurons/vestibular region/tissue section (Holstein et al., 2012). Given the higher sensitivity of the peroxidase labeling system, fewer non-stimulation-dependent cFos-positive vestibular neurons are likely to be obtained using the immunofluorescence approach taken in the present study.

The FluoroGold tracer was amplified by immunostaining with a rabbit polyclonal anti-FluoroGold serum (Millipore; Cat.# AB153). Both the c-Fos and FluoroGold primary antibodies provided staining of the same vestibular regions and cells observed previously (Holstein et al., 2011, 2012, 2014). Pha-L was visualized using a polyclonal rabbit anti-Pha-L (E + L) serum (Vector Labs). Both monoclonal and polyclonal antibodies against VGluTs-1 and -2 were used; VGluT-3 was identified using a rat monoclonal antibody only.

The GABA and glutamate antibodies are both mouse IgG1 monoclonals produced in our laboratory. Full descriptions of the production, characterization and specificity of the two antibodies were previously published (Holstein et al., 2004). Additional assays verified that glutamate can be fixed *in situ* by glutaraldehyde and that the fixed glutamate can specifically be recognized by the monoclonal antibody (MAb 215B2; Holstein et al., 2011).

#### Immunoperoxidase/Diaminobenzidine Staining

To identify the FluoroGold injection sites in the ventrolateral medulla or the Pha-L injection sites in the caudal VNC, series’ of vibratome sections from each injected rat were incubated in blocking buffer (PBS with 10% normal goat serum, 0.02% NaN_3_ and 0.1% Triton X-100) for 4–6 h or overnight, and then in anti-FluoroGold antibody (1:5000 in blocking buffer) or anti-Pha-L antibody (1:1000 in blocking buffer) for 12–18 h. After thorough rinsing (six changes of PBS over 4–6 h), the free-floating sections were incubated in peroxidase-conjugated goat anti-rabbit secondary antibody (Jackson ImmunoResearch Cat. # 111-035-144; 1:2000 in blocking buffer without NaN_3_) overnight. Sections were then rinsed in PBS (six changes over 2 h) and incubated in diaminobenzidine (DAB; 0.5 mg/ml; Sigma D-5905; St. Louis, MO, USA) diluted in 0.1M Tris buffer (pH 7.6) with 0.01% H_2_O_2_ for 5–10 min at room temperature. The reaction was stopped by repeated PBS rinses. Concomitantly processed control sections were treated as above, save for the omission of primary and/or secondary antibody.

#### Immunofluorescence

Tissue sections were processed for three-channel immunofluorescence detection of combinations of primary and secondary reagents. Some sections were also stained with DAPI (ThermoFisher Scientific; Cat # D-1306) to visualize the location and approximate size range of cell nuclei. Since both the cFos and FluoroGold primary antibodies were raised in rabbit, the studies were performed by sequential application of primary and secondary antibodies. Briefly: (i) sections were exposed initially to rabbit anti-cFos primary followed by fluorochrome-labeled (e.g., AlexaFluor 488) Fab fragment of a goat anti-rabbit IgG secondary; (ii) unreacted sites on the anti-cFos were blocked using a high concentration of *unlabeled* Fab fragment of goat anti-rabbit secondary antibody; and (iii) stained sections were exposed to rabbit anti-FluoroGold followed by goat anti-rabbit IgG secondary antibody tagged with a different fluorochrome (e.g., AlexaFluor 568). The sequence of primaries was alternated across staining experiments. A detailed description of the procedure is provided below.

All steps were performed at room temperature with agitation on an orbital shaker. Free-floating sections were blocked for 3–6 h in blocking buffer; incubated for 12–18 h in rabbit anti-c-Fos primary antibody (1:500 in blocking buffer); washed for 4–8 h with multiple changes of PBS; incubated for 12–18 h in AlexaFluor- or DyLite-conjugated goat anti-rabbit IgG (H + L) antibody Fab fragment (8 μg/ml in blocking buffer; Jackson ImmunoResearch); washed for 4–8 h in multiple changes of PBS; fixed briefly (10 min) with 2% paraformaldehyde; washed again; exposed 12–18 h to unlabeled Fab fragment goat anti-rabbit IgG (20 μg/ml in blocking buffer; Jackson ImmunoResearch); washed; treated 12–18 h with a mixture of rabbit anti-FluoroGold primary antibody (1:400 in blocking buffer) and mouse anti-glutamate (1:10 hybridoma supernatant in blocking buffer); washed; and treated with AlexaFluor-conjugated goat anti-rabbit IgG (H + L; 1:400 in blocking buffer; Invitrogen) and AlexaFluor-conjugated goat anti-mouse IgG (H + L; 1:300 in blocking buffer; Invitrogen). Following this staining, sections were incubated in DAPI solution (300 nM in PBS) for 30 min. After final washes, all sections were mounted on glass slides and coverslipped using Prolong Gold AntiFade mounting medium (Invitrogen). Since we have found varying degrees of sensitivity among secondary antibodies, several different secondary antibodies and several alternative fluorochromes (in different sections) were used to detect each primary antibody.

All experiments included the following controls: (1) tissue sections labeled with multiple secondary antibodies but only one primary antibody; (2) sections labeled with multiple secondary antibodies but no primary antibody; and (3) sections treated with the blocking and rinsing steps, but no antibody incubations. All data for this study derived from experiments in which each secondary antibody bound exclusively to its appropriate primary antibody and there was no binding to the inappropriate primary reagents. In addition, all of the immunofluorescence-stained sections contained profiles that were stained by only one of the colors in the specimen’s secondaries mixture. This provided further assurance within each individual section that only one secondary antibody bound to each primary antibody and therefore that secondary antibody cross-reactivity was insignificant.

#### Microscopy and Image Preparation

Sections were examined and images were acquired with a Zeiss Axioplan2 microscope equipped with structured illumination (ApoTome). Regions of interest were recorded with 10× or 20× objectives for cell body analysis, and 40× or 100× objectives for boutons. With our equipment, these yield image pixel sizes of 0.64, 0.32, 0.16, and 0.07 μm, respectively. Excitation and exposure conditions were kept constant within each image recording session.

Each session included images of intensity-calibrated fluorescent beads (InSpeck, Invitrogen), which allowed the intensity values in the images to be transformed to a scale based on the beads and therefore reasonably independent of image recording conditions. This strategy is advantageous because it permits comparisons of fluorescence intensities across specimens, experiments and imaging sessions. Using this approach, the level of fluorescence designated as “intense” was set at 75% of maximal fluorescence intensity.

Immunofluorescence was recorded using multichannel *z*-stacks (gray scale). The images were analyzed by assessing the immunofluorescence present in each gray scale optical slice of each single channel separately. Antibody co-localization was initially screened using maximum intensity projections (MIP) through the *z*-stacks and both *x-z* and *y-z* sideviews of pseudocolored image stacks constructed using the Zeiss Apotome software. Co-localization was then verified by comparing the gray scale immunofluorescence present in each individual channel within the same optical slice. Volocity, ImageJ and AxioVision were used to generate *z*-axis MIPs of whole stacks or selected subsets of optical sections for presentation figures. Images were prepared (cropped, sized, colored, labeled, annotated) using Adobe Photoshop and Illustrator CS6. Adjustments of brightness and contrast were performed using the Photoshop Levels and Curves tools applied to all parts of each image. The details of our image processing strategy for figure preparation are published in Holstein et al. (2014).

#### Atlas Data

Anatomical landmarks identified in all stained sections were used to estimate Bregma levels according to a standard rat brain stereotaxic atlas (Paxinos and Watson, 2009). As noted above, there were no significant differences in the caudal vestibular nuclei, RVLM or CVLM of the Wister-based atlas and our Long-Evans tissue sections. DAB stained sections used to identify the FluoroGold and Pha-L injection sites were imaged, and the location of the injection site and tracer diffusion penumbra in each animal were plotted manually on brainstem drawings referenced to the atlas sections. Similarly, all cFos- and FluoroGold-immunofluorescent double-labeled vestibular neurons were imaged and mapped. Data from the ten rats with FluoroGold injections within RVLM were pooled, as were the data from the seven rats with FluoroGold injections in CVLM.

#### Quantitation

Sections from the entire rostro-caudal extent of the vestibular nuclear complex were examined and fields containing cells positive for FluoroGold, cFos, and glutamate or GABA were recorded using 10× or 20× objective lenses. All FluoroGold-positive/cFos-positive cells were counted in images from immunofluorescence-stained sections containing at least one double-labeled vestibular neuron. Sections from the same animal were separated by at least 100 μm to avoid counting the same neuron twice.

#### Statistics

Data were analyzed using Microsoft Excel. One- and two-way contingency tables of pooled raw counts and congruent tables of expected values were constructed and chi-square analyses were performed using the Excel function chisq.test. Tests were not performed where any expected value was <5. Since comparisons were few, no correction for multiplicity of tests was performed.

## Results

**Figure 1:**
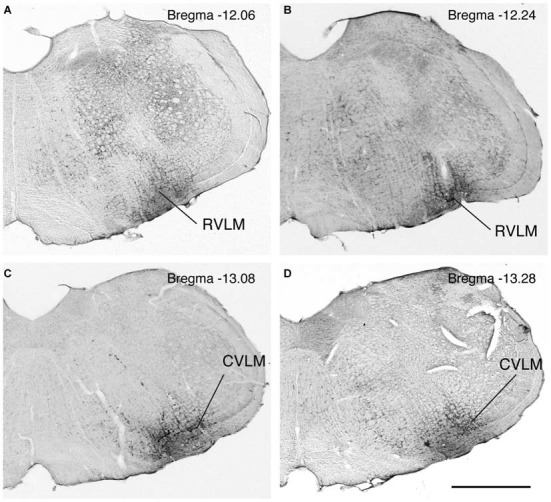
**Photomicrographs of immunoperoxidase/DAB-stained Vibratome sections from four rats, two with FluoroGold tracer injections in RVLM (A,B) and two with tracer injections in CVLM (C,D).** Bregma levels were estimated by matching anatomical boundaries with a stereotaxic atlas (Paxinos and Watson, 2009 #2695). Scale bar in lower right: 1 mm for all panels.

The rostro-caudal extent of the FluoroGold deposit in each of the 17 rats with a regionally specific injection is presented in Table 2 and examples of these injections are shown in Figure 1. In general, the injection sites and surrounding penumbrae extended less than 1 mm medio-laterally and less than 750 μm dorso-ventrally.

**Table 2:** **FluoroGold injection sites**.

	Caudal	Center	Rostral
**RVLM**
R600	−12.60	−12.30	−12.00
R601	−12.24	−12.12	−12.00
R647	−12.60	−12.36	−12.00
R669	−12.78	−12.48	−11.80
R652	−12.80	−12.40	−11.88
R687	−12.28	−11.90	−11.78
R696	−12.57	−12.11	−11.88
R698	−12.66	−12.43	−12.12
R700	−12.60	−12.36	−12.18
R701A	−12.84	−12.36	−12.12
**CVLM**
R609	−13.65	−13.08	−12.80
R688	−13.66	−13.41	−13.16
R690	−13.68	−13.43	−12.93
R695	−13.56	−13.22	−12.30
R697	−13.44	−13.20	−12.96
R702	−13.68	−12.96	−12.80
R709	−13.60	−13.44	−12.96

*Legend: Approximate Bregma levels of the tracer injection site (Center) and the rostral and caudal extent of the diffusion penumbra in RVLM or CVLM of all rats used for the multiple label immunofluorescence studies of cFos- and FluoroGold-positive neurons*.

FluoroGold-filled perikarya were observed in the caudal VNC of all 17 rats with tracer injections restricted to RVLM or CVLM. As previously observed (Holstein et al., 2011), most of the retrogradely-filled vestibular cells were located in the caudal half of SpVN, MVN and MVNpc. These cells, which had fusiform, multipolar, or small globular perikarya, were interpreted as vestibular neurons with direct projections to the injected region.

**Figure 2:**
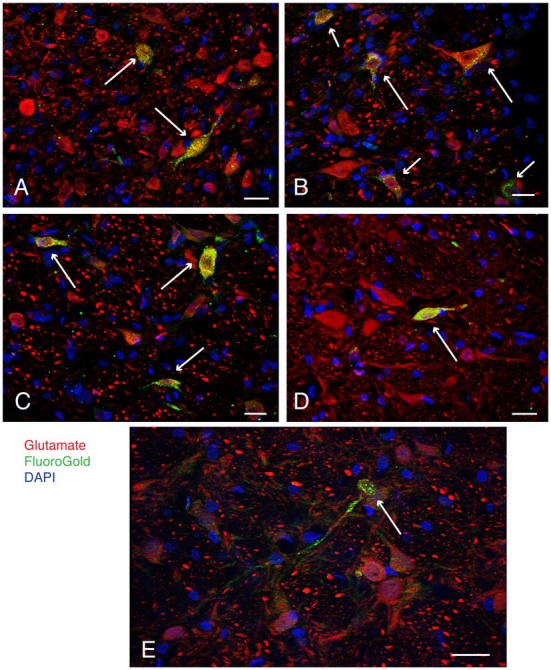
**Examples (long arrows) of intensely glutamate immunofluorescent neurons (red) in the caudal vestibular nuclei retrogradely-filled (green) following FluoroGold tracer injections in the ventrolateral medulla.** DAPI (blue) was used to visualize the location and approximate size range of cell nuclei. **(A)** Two neurons in caudal MVN, a small globular neuron above, and a fusiform cell below. Both neurons projected to contralateral RVLM. **(B)** A small globular neuron and a larger multipolar neuron in SpVN, both glutamate immunofluorescent and retrogradely filled with FluoroGold, projecting to ipsilateral RVLM. Shorter arrows point to three additional glutamate immunofluorescent and FluoroGold-filled neurons in the field. **(C)** Three FluoroGold-labeled glutamate immunofluorescent neurons in SpVN with direct projections to contralateral RVLM. **(D)** A glutamate immunofluorescent and FluoroGold-filled fusiform neuron in SpVN with terminals in contralateral RVLM. **(E)** A small glutamate immunofluorescent and FluoroGold-immunolabeled globular neuron in SpVN projecting to contralateral CVLM. All scale bars: 20 μm.

Selected vibratome sections through the caudal vestibular nuclei were processed for immunofluorescence detection of FluoroGold and glutamate. The low-affinity monoclonal anti-glutamate antibody used in this study was described in detail previously (Holstein et al., 2004, 2011). While some neurons had barely detectable immunofluorescence signal, a subset of neurons were intensely immunofluorescent. Only these latter cells were designated as glutamate-immunofluorescent neurons in the present study. All three cytological types of neurons in the caudal vestibular nuclei that were previously identified by retrograde tracing alone (multipolar neurons, fusiform neurons and small globular neurons) displayed co-localization of FluoroGold and glutamate immunofluorescence (Figure 2). Thus, aside from the larger diameters of most SpVN cells, there were no differences in the cytology of double-labeled FluoroGold and glutamate-immunofluorescent neurons in different vestibular regions (SpVN, MVN or MVNpc), in those projecting to RVLM vs. CVLM, or in the double-labeled neurons projecting to the ipsilateral vs. contralateral ventrolateral medulla.

**Figure 3:**
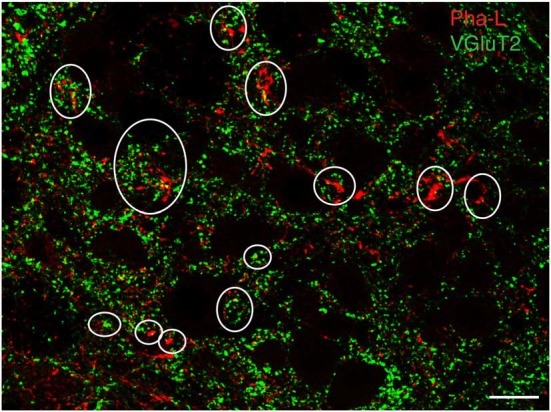
**Vesicular glutamate transporter 2 (VGluT2) in vestibular processes and terminals in the RVLM.** The vestibular axons were identified by anterograde Pha-L tracer injection in SpVN. Some PhaL-immunofluorescent vestibular processes and puncta (red) co-localize VGluT-2 (green). Some examples are encircled. Scale bar: 20 μm.

To support our hypothesis that the glutamate immunofluorescent neurons utilize this amino acid for neurotransmission, brainstem sections from three rats with anterograde Pha-L injections in the caudal vestibular nuclei were immunolabeled with antibodies to the three vesicular glutamate transporters (VGluTs), which are thought to be highly specific for glutamatergic neurotransmission (Fremeau et al., 2004; El Mestikawy et al., 2011). We did not observe co-localization of VGluT-1 or VGluT-3 in Pha-L-filled axons in RVLM or CVLM. However, many of the Pha-L labeled vestibular processes and terminals in RVLM and CVLM co-localized VGluT-2 (Figure 3), supporting the observations obtained using anti-glutamate immunolabeling, suggesting that a portion of the VSR pathway is glutamatergic.

**Figure 4:**
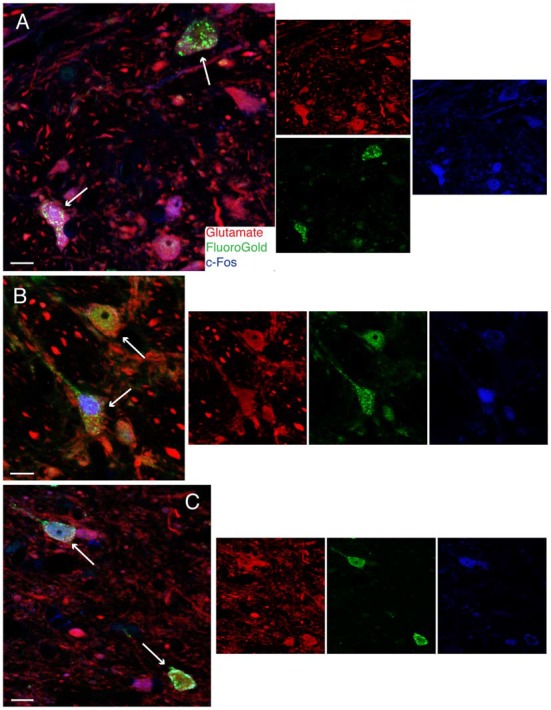
**Vibratome sections stained concomitantly for glutamate (red), FluoroGold (green) and cFos (blue) following tracer injections in the ventrolateral medulla and subsequent activation of the vestibulo-sympathetic reflex (VSR) using sinusoidally modulated GVS (sGVS).** Each large panel illustrates two activated projection neurons (arrows) with glutamate immunofluorescence in a merged image; the smaller panels to the right are the single channel images. **(A)** SpVN neurons with projections to contralateral RVLM. **(B)** MVN cells with projections to contralateral CVLM. **(C)** MVN neurons with projections to ipsilateral CVLM. Color key in **(A)** is for all panels. Scale bars in **(A–C)**: 10 μm.

To confirm the putative role of FluoroGold-labeled vestibular neurons in mediating the VSR, low frequency binaural sGVS was used to stimulate vestibular nerve fibers in all rats prior to perfusion. Activation of neurons in the vestibular nuclei was assessed by immunofluorescence detection of c-Fos protein. Some tissue sections from these animals were immunostained concomitantly for c-Fos, FluoroGold, and glutamate, in order to localize the amino acid in cells that were both activated by vestibular stimulation and had direct projections to one of the target regions (RVLM or CVLM). We refer to such cells as “activated projection neurons” of the VSR. Many of the sGVS-activated vestibular neurons with projections to either CVLM or RVLM were glutamate-immunofluorescent. Triple-labeled cells were present in both SpVN and MVN, and all three cytologic types were observed (Figure 4).

**Figure 5:**
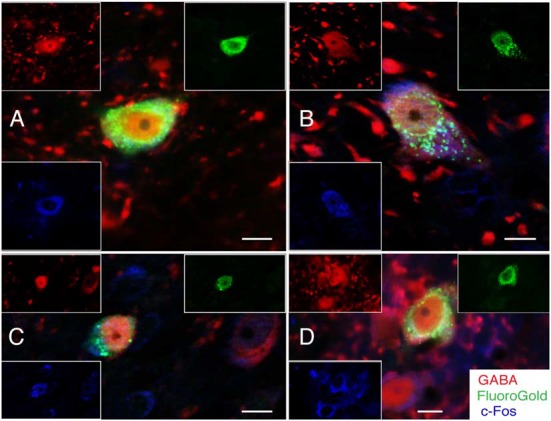
**FluoroGold-filled (green), sGVS-activated (cFos; blue) GABA-immunofluorescent (red) vestibular neurons. (A)** SpVN neuron with ipsilateral projections to CVLM. **(B–D)** MVN neurons with projections to contralateral CVLM. Each panel contains a central merged image with the three single channel images surrounding it. GABA-immunofluorescent processes and puncta were often observed adjacent to the GABA-immunofluorescent activated projection neurons **(A,B,D)**. Color key in **(D)** is for all panels. Scale bars in all panels: 10 μm.

Unexpectedly, we also found that some activated projection neurons in MVN and SpVN were intensely GABA-immunofluorescent (Figure 5). Aside from neurotransmitter content, the GABA-immunofluorescent activated projection neurons were indistinguishable cytologically from the glutamate immunofluorescent subpopulation. However, GABA-immunofluorescent processes and puncta were usually observed adjacent to the GABAergic activated projection neurons, suggesting substantial inhibitory input to these GABAergic VSR neurons.

### Quantitative Estimates of Glutamate- and GABA-Immunofluorescent VSR Neurons

As described above, representative tissue sections through the caudal vestibular nuclei of each rat with a unilateral FluoroGold tracer injection restricted to RVLM or CVLM were immunolabeled to visualize the tracer, cFos protein, and either glutamate or GABA. These data were used to obtain quantitative estimates of the relative frequencies of different subpopulations of glutamate- and GABA-immunofluorescent activated projection neurons. However, since glutamate and GABA were not co-localized in individual tissue sections (see “Materials and Methods” Section), and it is possible that some neurons could utilize both amino acids for neurotransmission, the data were not appropriate for estimating the total numerical populations of GABA- and glutamate-immunofluorescent VSR neurons.

### Glutamate-Immunofluorescent VSR Neurons Projecting to RVLM

One hundred twelve FluoroGold and cFos double-labeled vestibular neurons were examined in tissue sections further stained for glutamate immunofluorescence from rats with tracer injections in RVLM. Of the 112 cFos- and FluoroGold-positive neurons, 93 (83%) were glutamate-immunofluorescent (Table 3A). These triple-labeled neurons were more prevalent in SpVN than in MVN (57% vs. 43% of the total population of glutamate-immunofluorescent cells with direct projections to RVLM). Although glutamate-immunofluorescent VSR neurons projected to contralateral as well as ipsilateral RVLM, cells with ipsilateral projections were significantly more numerous (64% vs. 36%, respectively; *p* = 0.0007). Of the total population of glutamate-immunofluorescent VSR neurons projecting to RVLM, the largest cluster of cells was located in SpVN and projected ipsilaterally, the smallest subpopulation was located in SpVN and projected contralaterally, and the projections from MVN to ipsilateral and contralateral RVLM had intermediate values. Thus, while glutamate-immunofluorescent cells with direct projections to RVLM arise from both SpVN and MVN, and issue both ipsilateral and contralateral projections, the input to RVLM from ipsilateral SpVN is more than double that from the contralateral SpVN. In contrast, there is no statistically significant laterality predominance in the glutamate-immunofluorescent projections from MVN to RVLM.

**Table 3:** **Glutamate-immunofluorescent activated projection neurons in the caudal vestibular nuclei**.

	Ipsilateral (%)	Contralateral (%)	All (%)
**(A) Cells projecting to RVLM (*N* = 93)**
MVN	24	19	43
SpVN	40**	17**	57
Total	64*	36*	100
**(B) Cells projecting to CVLM (*N* = 62)**
MVN	18	6.50	24***
SpVN	48	27.50	76***
Total	66****	34****	100

Notes: *Significant difference by chisquare analysis, p = 0.0007; **Significant difference by chisquare analysis, p = 0.004; ***Significant difference by chisquare analysis, p < 0.0001; ****Significant difference by chisquare analysis, p = 0.01.

### Glutamate-Immunofluorescent VSR Neurons Projecting to CVLM

Seventy four FluoroGold and cFos double-labeled somata with direct projections to CVLM were observed in tissue sections through the caudal vestibular nuclei that were also immunostained for glutamate (Table 3B). Of these, 62 (84%) were triple-labeled. As with the RVLM-projecting neurons, glutamate-immunofluorescent VSR neurons projecting to CVLM were found in caudal SpVN and MVN, but were significantly more numerous in SpVN (76% vs. 24%; *p* < 0.0001) and more prevalent on the side ipsilateral to the tracer injection (66%; *p* = 0.01).

### GABA-Immunofluorescent VSR Neurons Projecting to RVLM and CVLM

As illustrated above, some activated projection neurons were GABA-immunofluorescent. The overwhelming majority of these (80%) projected to CVLM (Table 4). In addition, although they were present in both SpVN and MVN, over 40% were observed in SpVN and had ipsilateral projections (*p* = 0.02; Table 4B).

**Table 4:** **GABA-immunofluorescent activated projection neurons in the caudal vestibular nuclei**.

	Ipsilateral	Contralateral	All
**(A) Cells projecting to RVLM (*N* = 10)**			
MVN	2	0	
SpVN	7	1	
**(B) Cells projecting to CVLM (*N* = 39)**
MVN	13%*	28%*	41%
SpVN	41%**	18%**	59%
Total	54%	46%	100%

*Significant difference by chisquare analysis, *p* = 0.02; **Significant difference by chisquare analysis, *p* = 0.02.

**Figure 6:**
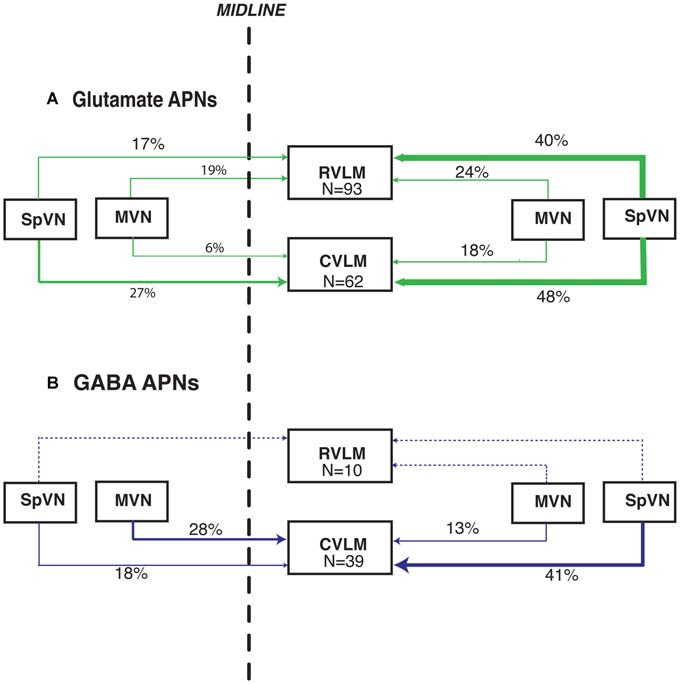
**Summary schematic diagram of the patterns of glutamate immunofluorescent (A) and GABA-immunofluorescent (B) sGVS-activated vestibular neurons with projections to RVLM and CVLM.** In this summary, the percentages of activated projection neurons in each of the four source regions (ipsilateral and contralateral SpVN and MVN) are calculated separately for projections to RVLM and to CVLM (The 99% total for glutamate-positive projections to CVLM is rounding error). Data for the triple-labeled GABA cells were obtained from eight rats; data for the triple-labeled glutamate-immunofluorescent cells were obtained from 12 rats. The line thickness reflects the relative proportion of those percentages. Dashed lines, which also lack percentage estimates, indicate the extremely small GABAergic projection to RVLM. This schematic highlights the putatively excitatory projection to ipsilateral RVLM and putatively inhibitory projections to ipsilateral and contralateral CVLM from the caudal vestibular nuclei.

Overall, these data demonstrate that glutamate immunofluorescent VSR neurons projecting to RVLM or CVLM are present in SpVN and MVN and as a population, give rise to both ipsilateral and contralateral pathways. In addition, there is a GABAergic VSR projection, also arising from both SpVN and MVN, that is almost exclusively directed to CVLM. These projections are summarized in Figure 6.

### The Rostro-Caudal Distribution of Glutamate- and GABA-Immunofluorescent VSR Neurons

The approximate Bregma level of each stained Vibratome section was identified based on landmark structures in the sections compared to a rat stereotaxic atlas (Paxinos and Watson, 2009). The sections, which extended overall from Bregma −13.30 to −10.45, were then binned into 10 representative levels: −13.08 (sections between −13.30 and −12.97), −12.84 (sections between −12.96 and −12.73), −12.60 (sections between −12.72 and −12.49), −12.36 (sections between −12.48 and −12.25), −12.12 (sections between −12.24 and −12.01), −11.88 (sections between −12.00 and −11.77), −11.64 (sections between −11.76 and −11.53), −11.40 (sections between −11.52 and −11.29). −11.16 (sections between −11.28 and −10.99) and −10.8 (sections between −10.98 and −10.45). The cells were mapped, distinguishing ipsilateral from contralateral projections to RVLM and CVLM, with the simplifying assumption that all activated projection neurons had exclusively ipsilateral or contralateral projections.

**Figure 7:**
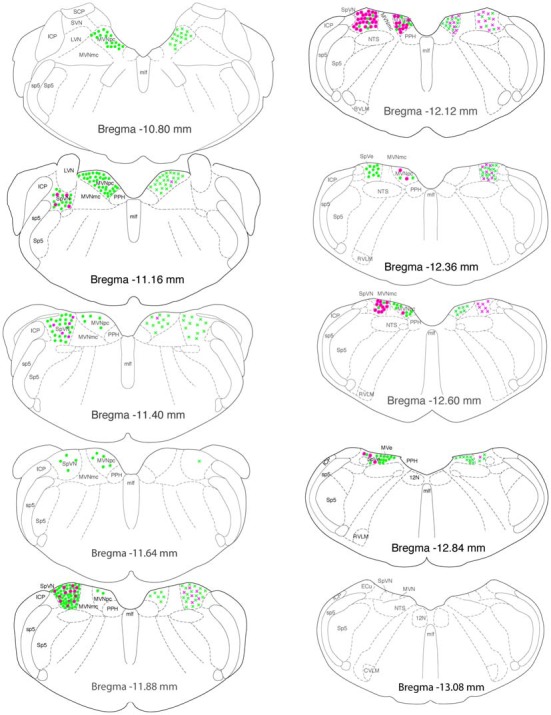
**Location of glutamate immunofluorescent vestibular neurons that were activated by sGVS and had direct projections to the ipsilateral (filled circles on left side of each drawing) or contralateral (Xs on right side of each drawing) RVLM (green) or CVLM (magenta).** There were two high density clusters of glutamate immunofluorescent activated projection neurons, one located at Bregma −11.16 and one at Bregma −11.88. The highest overall density of glutamate immunofluorescent activated vestibular neurons with projections to CVLM was observed at Bregma −12.12. The individual drawings are based on Paxinos and Watson (2005). Abbreviations not used in the text previously: 12 N, hypoglossal nucleus; ICP, inferior cerebellar peduncle; LVN, lateral vestibular nucleus; MLF, medial longitudinal fasciculus; MVNmc: magnocellular region of MVN; MVNpc, parvocellular region of MVN; NTS, solitary nucleus; PPH, prepositus nucleus; SCP, superior cerebellar peduncle; sp5, spinal trigeminal tract; Sp5, spinal trigeminal nucleus; SVN, superior vestibular nucleus.

Glutamate-immunofluorescent activated projection neurons were observed throughout all but the caudal-most pole of the vestibular nuclear complex (Figure 7). Both ipsilaterally- and contralaterally-projecting cells were found from Bregma −12.96 through −10.54. However, cells with direct projections to the ipsilateral RVLM were concentrated in two regions: SpVN at approximately Bregma −11.88 and parvocellular MVN near Bregma −11.16. In contrast, glutamate-immunofluorescent VSR neurons projecting to ipsilateral CVLM were concentrated more caudally, near Bregma −12.12. Similarly, cells projecting to contralateral RVLM were widely dispersed through the caudal vestibular nuclear region, whereas those projecting to contralateral CVLM were concentrated caudally, and primarily in SpVN.

**Figure 8:**
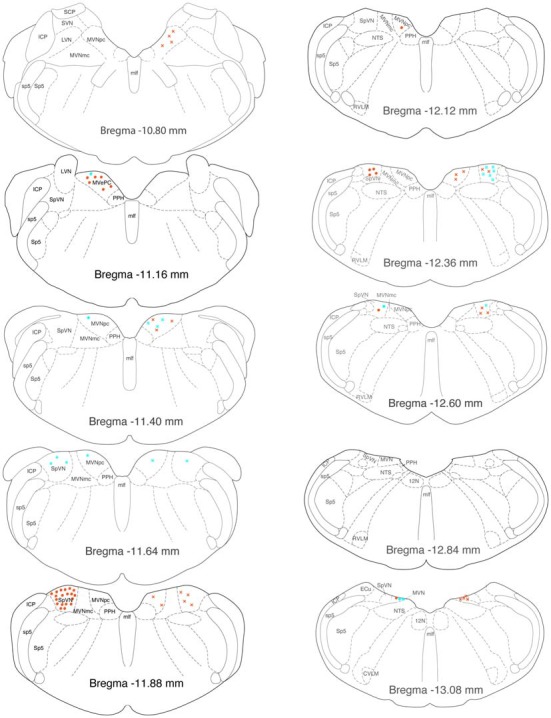
**Location of GABA-immunofluorescent vestibular neurons that were activated by sGVS and had direct projections to the ipsilateral (filled circles on left side of each drawing) or contralateral (Xs on right side of each drawing) RVLM (aqua) or CVLM (orange).** The highest overall density of these cells was observed at Bregma −11.88. The preponderance of GABA-IMF activated vestibular neurons project to CVLM, not RVLM. The individual drawings are based on Paxinos and Watson (2005). Abbreviations as in Figure 7.

GABAergic activated projection neurons were observed from Bregma −13.18 to −10.94 (Figure 8). Most of these neurons projected from SpVN to the ipsilateral CVLM and these cells were most highly concentrated near Bregma −11.88.

## Discussion

### Glutamate and GABA in Central Vestibular Neurons

This study found intense glutamate immunofluorescence in vestibular neurons that are activated by sGVS and project to presympathetic regions of the ventrolateral medulla. Since glutamate is present in the cytoplasm of all neurons as part of the metabolic store of amino acids, these observations do not definitively identify glutamate as a neurotransmitter of the VSR. To supplement the findings obtained using the low affinity anti-glutamate antibody, three glutamate transporters (VGluT1–3) were localized in sections through the ventrolateral medulla containing anterogradely-filled vestibular axons and terminals. These studies demonstrated co-localization of VGluT-2, but not VGluT-1 or -3, in vestibular processes and endings in the RVLM and CVLM, supporting the proposition that many vestibulo-sympathetic pathway neurons utilize glutamate for neurotransmission. However, since VGluT-2 has been identified in medullary neurons that manifest a non-glutamatergic phenotype, and since there is a lack of correspondence between glutamate and VGluT mRNA-containing somata in this region (Stornetta et al., 2002a,b; Llewellyn-Smith et al., 2007), the presence of the VGLUT-2 glutamate transporter in vestibular terminals must also be interpreted conservatively. Thus, our data suggest, but do not definitively demonstrate, that some portion of the VSR is glutamatergic.

Transmitter levels of glutamate or aspartate have previously been described in all four main vestibular nuclei (Kevetter et al., 2000; Holstein, 2012; Vidal et al., 2015) and have been associated with several different vestibular effector pathways (Highstein and Holstein, 2006). Subregions of the vestibular nuclei send glutamatergic projections to contralateral abducens motor and internuclear neurons, mediating the excitatory limb of the horizontal vestibulo-ocular reflex. Similarly, central vestibular neurons of the vertical vestibulo-ocular reflex send glutamatergic projections to the contralateral trochlear and oculomotor cell groups and at least a portion of the lateral vestibulo-spinal tract, which conveys otolith signals to the extensor motor neurons of the spinal cord, utilizes glutamate (Kevetter and Coffey, 1991; Du Beau et al., 2012). Some vestibulo-cerebellar projections are glutamatergic, as are the excitatory fibers of the vestibular commissural system, which is important for bilateral vestibulo-ocular control. Thus, glutamate neurotransmission is widespread in the central vestibular nuclei, and would not in itself serve as a unique identifier of VSR pathway neurons.

This study also found that a small component of the VSR pathway is GABAergic. Like glutamate, however, GABA is not exclusively found in vestibular neurons with autonomic projections (Popratiloff and Peusner, 2011). GABAergic neurons in various parts of the vestibular nuclear complex send projections to oculomotor neuron pools, participate in mediating commissural inhibition (Holstein et al., 1999a; Straka and Dieringer, 2000), contribute to vestibulo-olivary (Barmack and Yakhnitsa, 2013) and vestibulo-spinal (Blessing et al., 1987) tracts, and may also comprise a population of local circuit neurons (Grassi et al., 1995; Holstein et al., 1999b). Although the GABAergic component of the VSR pathway appears to arise from a relatively small group of vestibular neurons, our previous studies have demonstrated that the VSR axons in RVLM and CVLM are highly branched and laden with varicosities (Holstein et al., 2011). These two structural features suggest that each of the GABAergic, as well as glutamatergic VSR neurons exerts widespread influence over large areas of the target nuclei.

The diverse functional roles of glutamate and GABA in vestibular effector pathways are supported by electrophysiological and biochemical studies that have provided direct evidence for GABAergic (Bagnall et al., 2007) and glutamatergic (Broussard, 2009) neurotransmission in the mouse vestibular nuclei. Moreover, the transmitter phenotypes of mouse MVN neurons were evaluated using single-cell expression profiling (Kodama et al., 2012). This latter study, which identified three excitatory and three inhibitory cell types (E1–3 and I1–3, respectively), was restricted to a portion of MVN located rostral to many of the vestibulo-sympathetic neurons, particularly those with projections to CVLM, and included both magnocellular and parvocellular MVN regions. The E1 and E2 groups of this study were both glutamatergic; E1 is thought to comprise vestibulo-ocular neurons with projections to contralateral oculomotor nuclei and E2 cells projecting primarily to cerebellar cortex. The role(s) of E3 glutamatergic cells were not identified, but the authors speculated that these cells may contribute to vestibulo-autonomic circuitry. Based on the topography of our glutamate-immunofluorescent activated projection neurons, it is likely that at least some of the more caudally located E3 cells are VSR pathway neurons. Among the inhibitory cells, the I1 group was predominantly glycinergic, and the I2 cells gave rise to the ipsilateral projection to the abducens nucleus (Spencer and Baker, 1992). Most cells in the I3 group of inhibitory neurons were interpreted as GABAergic, perhaps participating in the inhibitory component of the vestibular commissure. Based on the present findings, a portion of this I3 cell group may comprise the GABAergic cells of the VSR pathway.

### Cytology of Glutamate and GABA-Immunofluorescent Vestibulo-Sympathetic Neurons

Our previous studies have identified three cell types that send direct projections to the ventrolateral medulla. These include (a) multipolar neurons with 3–5 dendrites radiating from the soma, and cell bodies of 25–35 μm in equatorial diameter; (b) smaller spherical neurons approximately 10–15 μm in diameter, with fewer dendritic processes; and (c) spindle-shaped neurons with dendrites emanating from each pole of the cell body (Holstein et al., 2014). All three cell types were observed in the present study as well, and there was no correlation between the cytology of the neuron and its amino acid content, location in SpVN vs. MVN, terminal field in RVLM vs. CVLM, or laterality of the projection. However, none of the vestibulo-autonomic neurons were of the larger diameters characteristic of vestibulo-ocular and vestibulo-spinal cells. Thus, while GABAergic and glutamate-immunofluorescent vestibulo-sympathetic neurons cannot be distinguished from each other on the basis of cytology, they are markedly different in overall size from the majority of vestibular neurons.

### Regional Localization of Glutamate and GABA-Immunofluorescent Vestibulo-Sympathetic Neurons

Overall, vestibulo-sympathetic activated projection neurons were observed in caudal MVN, parvocellular MVN and the caudal half of SpVN. The glutamate-immunofluorescent activated projection neurons were present in both SpVN and caudal MVN, with the population of cells projecting to RVLM located slightly more rostrally than those projecting to CVLM. In contrast, the majority of GABA-immunofluorescent cells projecting to the ventrolateral medulla were concentrated in SpVN, and sent ipsilateral projections to CVLM. Since the neurons in this study were identified in part using sGVS activation, and sGVS primarily activates otolith afferents (Ray et al., 1998; Watson et al., 1998; Lau et al., 2003; Bent et al., 2006; Cohen et al., 2011b), it is not surprising that the distributions of VSR neurons identified in this study correspond well with otolith nerve projection patterns, which primarily target SpVN and caudal MVN (Dickman and Angelaki, 2002; Straka et al., 2002; Newlands and Perachio, 2003; reviews: Barmack, 2006; Angelaki and Cullen, 2008). Similarly, the suggestion of a topographic organization in the VSR cells may reflect a more general topographic organization in the vestibular nuclei. At least in MVN, the larger neurons in the magnocellular region receive afferents from the semicircular canals and the flocculus, send projections to the extraocular motor nuclei, and are functionally related to the vestibulo-ocular reflex, whereas neurons in the caudal and parvocellular regions tend to be smaller, receive inputs from the otolith organs, spinal cord, nodulus, uvula and possibly the fastigial nucleus, and send efferents primarily to the inferior olive, cerebellum, ventrolateral medulla, and spinal cord (Büttner-Ennever and Gerrits, 2004). It remains to be determined, however, whether vestibular neurons projecting to RVLM and/or CVLM are segregated from or intermingled with those projecting to other autonomic medullary regions such as the solitary nucleus and the dorsal motor vagal nucleus that are important for cardiac and gastrointestinal functions (Balaban and Beryozkin, 1994; Yates et al., 1994, 2014b; Ruggiero et al., 1996).

### Functional Significance

When humans rise from a supine position, or quadrupeds rear or climb, there is a caudal shift in blood and other body fluids. As blood pools in the leg vasculature, there is decreased venous return to the heart, which reduces cardiac output and blood pressure. Concomitantly, because of the change in body orientation, the force of gravity exerts a greater impact on the fluids in the lower body, requiring higher blood pressure to overcome that force than would be required if the body was horizontal (Hall, 2016). Blood redistribution due to a postural change is initially triggered by the VSR pathways from the caudal vestibular nuclei to presympathetic regions of the medulla, including the RVLM and CVLM (Yavorcik et al., 2009). In general, these pathways assure that postural adjustments do not cause marked fluctuations in cerebral perfusion, which would deprive the brain of a consistent supply of oxygen and glucose. Subsequent to the activation of sympathetic nerve activity by the VSR, the baro- and cardiopulmonary reflexes re-establish and maintain homeostatic blood pressure control (Yates and Miller, 1994; Yates and Bronstein, 2005).

**Figure 9:**
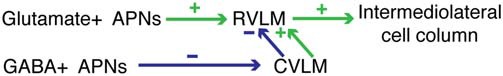
**One example of the hodology supported by the present data.** Differential activation of component parts of the pathway could result in either increased or decreased blood pressure. See text for details.

Activation of the VSR pathway in experimental animals has been achieved using a variety of physiological (Doba and Reis, 1974; Yates and Miller, 1994; Woodring et al., 1997; Jian et al., 1999; Mori et al., 2005; Wilson et al., 2006; Abe et al., 2007, 2008a; Yavorcik et al., 2009) and electrical (Cobbold et al., 1968; Uchino et al., 1970; Steinbacher and Yates, 1996; Kerman and Yates, 1998; Kerman et al., 2000a,b; Yates et al., 2000; Kasumacic et al., 2012) stimuli ranging from linear acceleration and centrifugation to electrical stimulation of the vestibular labyrinth or nerve. The resultant VSR-related activity has been assessed using measurements of physiological parameters such as blood pressure and heart rate, as well as recordings of sympathetic nerve activity associated with innervation of target tissues such as vascular smooth muscle and skin. Overall, these studies have demonstrated increased, decreased, and/or biphasic sympathetic responses to vestibular stimulation (Bolton et al., 2004; Gotoh et al., 2004; Abe et al., 2008b) leading to the summary conclusion that the role of the VSR is modulatory and complex (Yates et al., 2014a). While the results of the present study support this overall conclusion, the existence of GABAergic as well as putatively glutamatergic VSR projections may provide an additional/alternative explanation for the variability observed in response outcome following VSR activation. Conceivably, the magnitude and direction of blood pressure changes mediated by the VSR reflect the relative activation of two pathways—an excitatory projection to RVLM that utilizes glutamate and an inhibitory projection to CVLM that utilizes GABA. The latter cells, in turn, may activate excitatory and/or inhibitory neurons in CVLM that project to RVLM, adding further possible flexibility in the blood pressure responses to otolith activation (Figure 9). In its simplest configuration, contemporaneous stimulation of the glutamate VSR pathway to RVLM and the GABA VSR projection to GABAergic neurons in CVLM would result in activation of the maximal percentage of presympathetic neurons in the RVLM, resulting in a maximal VSR-induced increase in blood pressure. In contrast, predominant activation of only one of the two pathway components would diminish or even reverse this effect on blood pressure. Further subtleties in blood pressure changes could result from differential activation of the synergist parallel pathways, depicted in Figure 6 but not Figure 9. Additional studies will be needed to identify the specific roles of these two facets of the VSR.

### Methodological Considerations and Study Limitations

1. The quantitative estimates are based on triple-labeled cells only. To be included in the cell counts, individual neurons needed to display specific immunofluoresence signal for FluoroGold, cFos and one of the two amino acids examined in this study. Since these staining protocols may fail to triple-stain all relevant cells, and since imunofluorescence sensitivity is lower than that of immunohistochemistry, the cell counts obtained in this study are undoubtedly underestimates of the total populations. Furthermore, we observed much larger populations of double-labeled GABA and glutamate stained cells (e.g., cFos and GABA or FluoroGold and GABA immunofluorescent, but not cFos-, FluoroGold- and GABA-labeled) that were excluded from the study, although some of these cells may well be VSR neurons. For these reasons, the counts are likely to understate the real size of the total populations, although the relative proportions of the subpopulations are likely to stay the same.

2. Multiple additional neurotransmitters and modulators are thought to be present in VNC neurons. For example, immunolabeling and expression profiling studies suggest that some GABA- and glutamate-containing VNC neurons co-express glycine (Tanaka and Ezure, 2004; Lu et al., 2008; Popratiloff and Peusner, 2011; Kodama et al., 2012). However, the present study focused exclusively on the localization of glutamate and GABA in activated projection neurons of the VSR and did not include a more extensive assessment of the complete neurochemical phenotype of these cells.

3. Isoflurane inhalation anesthesia was used for all experiments of the present study. There is some evidence that *c-fos* gene expression is affected by general anesthetics, increasing gene activity in autonomic nuclei (Takayama et al., 1994; Hamaya et al., 2000; Roda et al., 2004). However, it appears that different anesthetics and different routes of administration impact *c-fos* gene activation and subsequent protein expression variably. Particularly pertinent to the present study, decreases in blood pressure, often observed with sGVS, can be accompanied by increased numbers of cFos-immunolabeled neurons in appropriate brainstem regions. Moreover, the number of cFos-positive neurons in limbic areas of the brainstem and cerebral cortex are no different in isoflurane anesthetized (3% induction, 2% maintenance) and unanesthetized rats (Kufahl et al., 2009). In addition, while *c-fos* mRNA expression in rat brain is significantly higher than baseline after 30 min of exposure to 2% isoflurane, the expression level returns to baseline within 60 min, even when the anesthesia is continued for a total of 2 h (Hamaya et al., 2000). Thus, isoflurane does not appear to alter *c-fos* gene transcription or depress *c-fos* mRNA expression. Based on these reports, and our observations in sGVS control rats (Holstein et al., 2012), it is unlikely that the cFos immunolabeling observed in the present study is attributable to the isoflurane anesthesia.

## Author Contributions

All authors approved the final version of this article. Experiments were conceived and designed by GRH, GPM and VLF. GPM performed the tracer injections; GPM and GRH performed the immunolabeling studies; GRH and VLF performed the microscopy and image processing; GRH and VLF performed the data analysis and all authors contributed to the manuscript and figure preparation.

## Funding

The research supported by the National Institute on Deafness and Other Communication Disorders grant #DC008846; NIH.

## Conflict of Interest Statement

The authors declare that the research was conducted in the absence of any commercial or financial relationships that could be construed as a potential conflict of interest.
